# Strain Echocardiography in Early Detection of Doxorubicin-Induced Left Ventricular Dysfunction in Children with Acute Lymphoblastic Leukemia

**DOI:** 10.5402/2012/870549

**Published:** 2012-01-24

**Authors:** Mohammed Al-Biltagi, Osama Abd Rab Elrasoul Tolba, Mohammed Ramadan El-Shanshory, Nagla Abd El-Aziz El-Shitany, Eslam El-Sayed El-Hawary

**Affiliations:** ^1^Pediatric Cardiology Unit, Tanta University, Tanta 31111, Egypt; ^2^Pediatric Hematology and Oncology Unit, Tanta University, Tanta 31111, Egypt; ^3^Department of Pharmacology and Toxicology, Faculty of Pharmacy, Tanta University, Tanta 31111, Egypt

## Abstract

*Objective*. To investigate the ability of two-dimensional longitudinal strain echocardiography (2DST), to detect the early doxorubicin cardiotoxicity. *Patients and Methods*. The study included 25 children with newly diagnosed acute lymphoblastic leukemia (ALL) aged 5–15 years and 30 healthy control children. They had echocardiographic examination with conventional 2-dimensional (2D), pulsed tissue Doppler (PTD), and 2DST echocardiography before and within 1 week after doxorubicin treatment. *Results*. There was no significant difference in left ventricle (LV) systolic and diastolic functions measured by conventional 2-D and PTD echocardiography between patients and controls. However, there was significant decrease in LV global and peak systolic strain detected by 2-DST echocardiography in study group than control. After doxorubicin treatment, there was no significant difference in LV systolic and diastolic functions measured by conventional 2-D and PTD echocardiography than before treatment except for prolonged IVCT and IVRT, but LV global and peak systolic strain was significantly lower after treatment. *Conclusion*. 2-D longitudinal strain echocardiography was more sensitive than conventional 2-D and PTD in detecting the early LV doxorubicin-induced cardiotoxicity in children with ALL.

## 1. Introduction

Doxorubicin is one of the most effective available anthracycline chemotherapeutic agents that have been widely used in the treatment of pediatric malignancies. Its introduction has led to the successful treatment of childhood cancer with improved survival rates. Nearly two-thirds of survivors have one or more related chronic medical problems and may require multidisciplinary care [1]. Because doxorubicin plays a key role in the treatment of many malignancies, its loss from the field of cancer treatments would lead to a dramatic reduction in cure rates. Its efficacy is often limited by its related cardiotoxicity, which leads to cardiomyopathy that may evolve into heart failure [2–4]. Cardiotoxicity can arise acutely, during or shortly after treatment and regardless of dose in the form of cardiac arrhythmias, for example, sinus tachycardia, ventricular, and supraventricular. Pericarditis, potentially fatal congestive heart failure, and acute pulmonary edema can also occur during doxorubicin therapy caused by myocyte necrosis as a result of increased cardiac apoptosis and alteration of cardiac cytochrome P450 expression and arachidonic acid metabolism [5, 6].

Chronic dose-related cardiotoxicity is the most common and dangerous and can manifest as cardiac failure, months or even years after the completion of treatment and is thought to be related to myocardial toxicity [7]. Multiple risk factors for doxorubicin-associated cardiotoxicity have enabled clinicians to define a high-risk population for cardiotoxicity. However, some low-risk patients can develop cardiotoxicity. On the other hand, not all high-risk patients develop cardiotoxicity [8].

The increase in prevalence of doxorubicin cardiotoxicity is partly due to improved diagnosis and partially owing to longer follow-up periods [9]. To detect cardiac damage, the adopted diagnostic approach depended mainly on the estimation of left ventricle ejection fraction (LVEF) or left ventricle fractional shortening (LVFS). Left ventricular systolic function (EF or FS) after doxorubicin therapy is generally assessed using M-mode and 2D echocardiography. On the basis of dimensional changes and volume calculations, FS and EF are calculated. This approach showed low sensitivity toward early prediction of cardiomyopathy; that is, why new modalities are being introduced into clinical trials. During the last decade, new echocardiographic techniques for evaluating myocardial function have been introduced.

The 2-dimensional strain echocardiography (2DSE) is a relatively new echocardiographic modality based on measurement of myocardial deformation using speckle-tracking from *B* mode images. Myocardial velocity and deformation imaging, namely, strain and strain rate imaging, have been demonstrated to have potential value for the quantification of global and regional systolic and diastolic myocardial function. It seems that regional dysfunction can be detected earlier than global dysfunction [10]. This might provide the rationale to start treatment of doxorubicin cardiotoxicity early in asymptomatic patients.

More studies are needed to define the best predictive parameters for those patients at risk of developing LV dysfunction who might benefit from an early start of treatment [11, 12]. The aim of the presenting work was to investigate the ability of echocardiography, especially the recent modalities as two-dimensional longitudinal strain echocardiography, to detect early cardiotoxic effect of doxorubicin received before and after the induction therapy in children with acute lymphoblastic leukemia (ALL).

## 2. Patients and Methods

A group of 25 children with newly diagnosed acute lymphoblastic leukemia between 5 and 15 years of age presented to the Hematology and Oncology Unit, the Pediatric Department, Faculty of Medicine, Tanta University, Egypt, who met the inclusion criteria, were included in the study from March 2008 to March 2010. Thirty healthy children of matched age and sex were studied as a control group.

### 2.1. Inclusion Criteria

Newly diagnosed children with acute lymphoblastic leukemia confirmed by complete blood picture, bone marrow examination, and immunophenotyping by flow cytometry and fluorescence in situ hybridization (FISH) technique.

### 2.2. Exclusion Criteria

Previous chemotherapy or radiotherapy, presence of any cardiac disease either congenital or acquired, any cardiac lesion detected in baseline echocardiography, any associated systemic disease that can affect the cardiac function, and/or medication that can affect cardiac function, such as angiotensin-converting enzyme inhibitors, angiotensin receptor blockers, diuretics, or beta-blockers.

All children subjected to full history taking, thorough clinical examination, complete blood picture (CBC), erythrocyte sedimentation rate (ESR), serum uric acid, and liver and renal function tests. All children underwent echocardiographic Doppler examination and measurement of troponin I (cTnI), and creatine phosphokinase CPK (MB) levels before and within 1 week of starting the doxorubicin treatment. All patients were subjected to the protocol of therapy in induction of remission which showed in Table 1.

**Figure 1:**
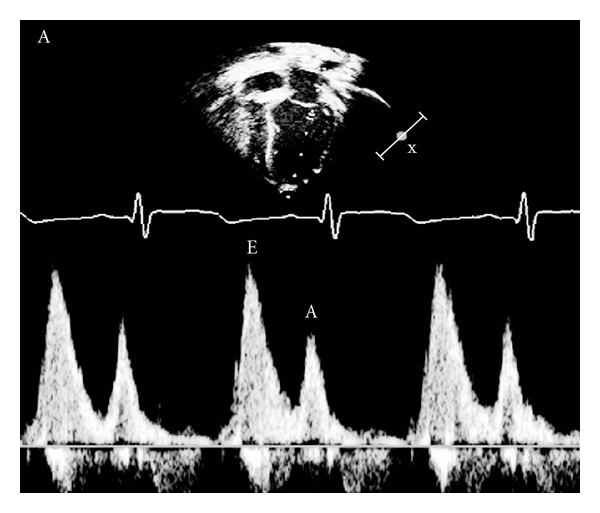
Pulsed-wave Doppler pattern of mitral inflow. It shows the peak velocities during early diastole (E) and atrial contraction (A).

Echocardiographic images were obtained using a Vivid 7 ultrasound machine (GE Medical System, Horten, Norway with a 3.5-MHz multifrequency transducer). To avoid intraobserver variability, 2 examinations each time were performed by the same operator for each patient in 2 different settings within 2 days. All children were examined in a semi-supine, left lateral position and according to the recommendation of the American Society of Echocardiography [13]. M-mode and two-D echocardiography were done to asses left ventricular (LV) internal dimensions, ejection fraction (EF), and fraction shortening (FS). Mitral flow early-phase filling velocity (E), peak atrial phase filling velocity (A), and E/A ratio were recorded by pulsed-wave Doppler in the apical 4-chamber view where the sample volume was best positioned in the left ventricle at the tips of the valve leaflets (distal to the annulus) (Figure 1).

### 2.3. Pulsed Tissue Doppler Image (TDI)

**Figure 2:**
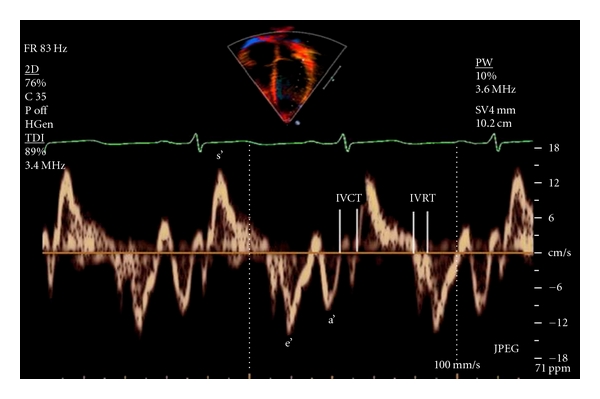
Lateral mitral annular tissue Doppler tracing. s′: peak velocity during ventricular systole; e′: peak velocity during early ventricular diastole; a′: peak velocity during atrial contraction; IVCT: isometric (isovolumic) contraction time; IVRT: isometric (isovolumic) relaxation time.

Pulsed tissue doppler imaging was done using pulsed wave DTI filters, with a sample volume 5.5 mm, and frame rates of greater than 150 fps were recorded. The baseline was adjusted to low-velocity range (−20 to 20 cm/s) with minimal gain setting. The sample volume was placed within the myocardium equidistant from the endocardial and epicardial borders. Effort was done to minimize the angles as much as possible. From the apical 4-chamber planes, using pulsed-wave TDI, the myocardial velocity curves of septal and lateral mitral valve annuli were recorded. The ECG was connected and traced simultaneously to define and to time the cardiac cycle events. The beginning of QRS complex was used as a reference point (Figure 2).

### 2.4. Velocities and Interval Measurements

The *s* wave reflects the systolic function of left ventricle (LV). The *e*′/*a*′ (early/atrial) ratio of mitral valve annulus reflects the diastolic function of LV. Isometric contraction time (IVCT) was defined as the time duration between the beginnings of QRS complex in the ECG to the beginning of DTI systolic (s) wave. Isometric relaxation time (IVRT) was defined as the interval between the end of *s* wave and the beginning of the *e*′ wave. At least 10 cardiac cycles were recorded from each site on a strip-chart recorder at a speed of 100 mm/s.

### 2.5. 2D Longitudinal Strain Echocardiogram Images

**Figure 3:**
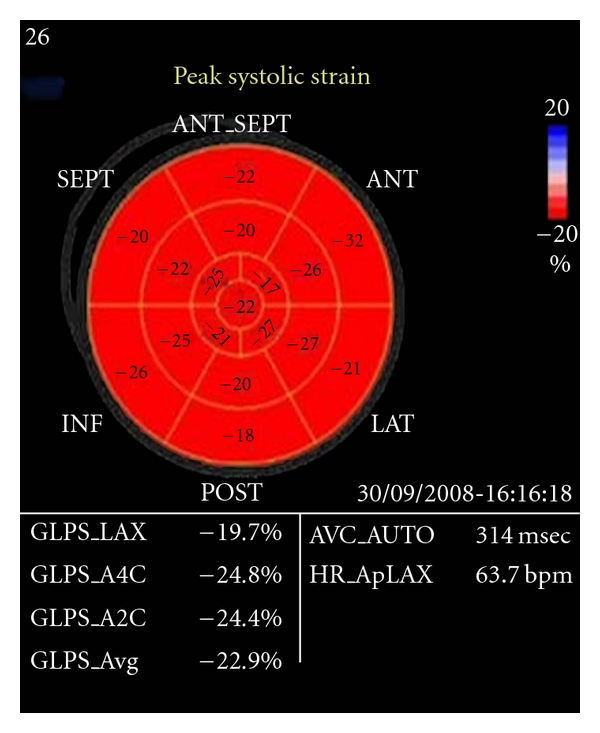
Figure 3: Eye bull projection for normal 2D strain (red colored) for one of the control group. GS = −22.9%.

**Figure 4:**
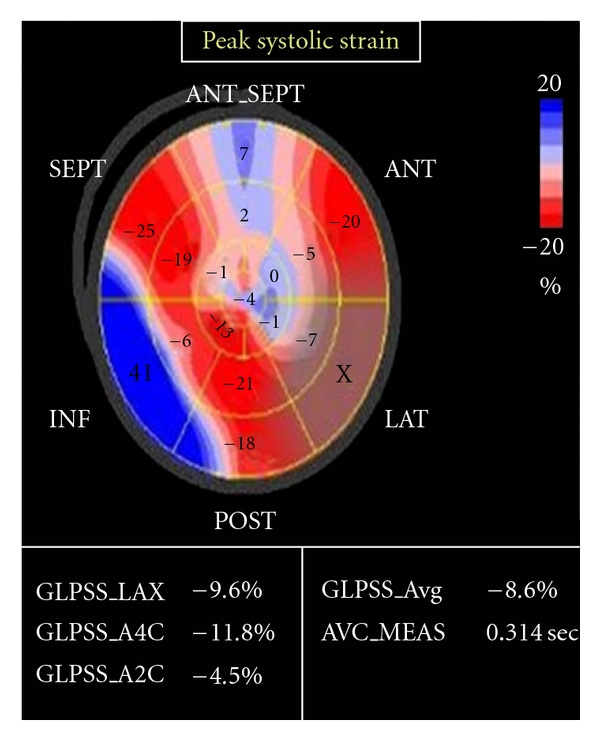
2D strain echocardiography in patient with doxorubicin-induced cardiotoxicity.

**Figure 5:**
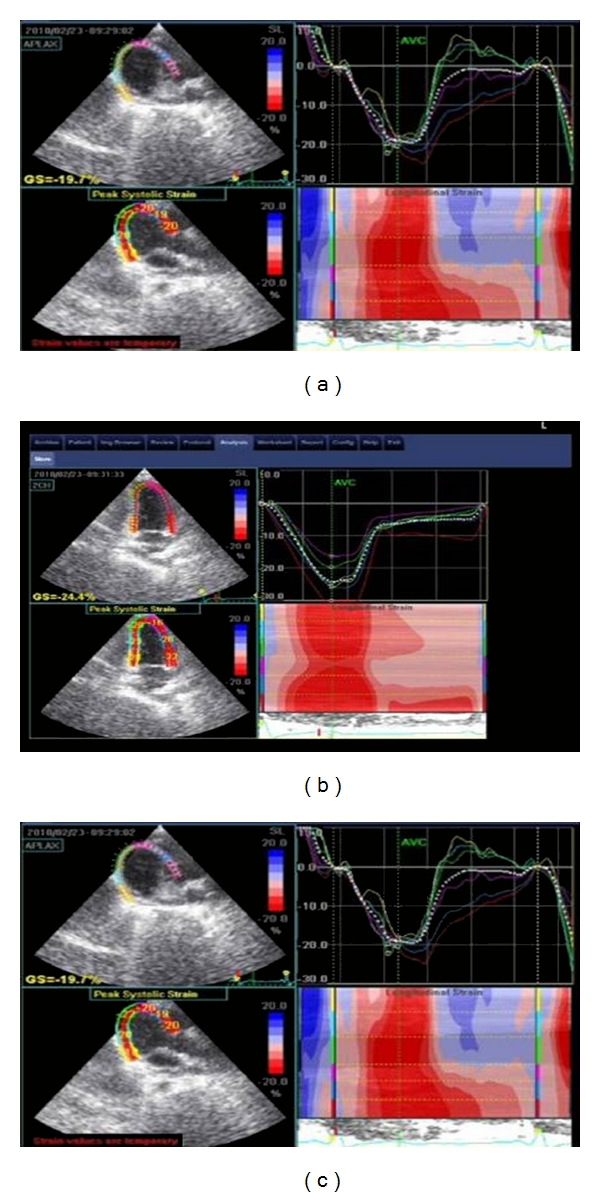
Examples of global longitudinal strain measures from the 3 standard apical views. Quad screen views from A4C (top), A2C (middle), and ALX (bottom: in each, the upper left quadrant shows tracking and also average peak strain for the segments measured (given as GS). Upper right quadrant shows color-coded segmental strain curves and average strain curve (dashed line). Bottom left quadrant graphically denotes peak strain in each segment. Lower right quadrant depicts anatomic M-mode.

Were obtained using the 3 standard apical views, apical long axis (ALX), apical 4-chamber, and apical 2-chamber views, and parameters obtained represented the average of 10 cardiac cycles, with a frame rate of 65 fps, and all segmental data (17 segments) were represented. We used automated function imaging that enables only the assessment of longitudinal strain available in Vivid 7 ultrasound machine to measure average LV global peak systolic strain (*G*), global peak systolic strain in 3 standard apical views, and segmental peak systolic strain in; basal, mid and apical segments of anteroseptal, anterior, lateral, posterior, inferior, and septal LV walls [10]. The images were transferred to the EchoPACS workstation with *Q* analysis software Version 4.0.3 (General Electric, Waukesha, WI, USA) for processing (Figures 3, 4, and 5).

Five milliliter of blood was transferred to plastic tubes to estimate cTnI and CPK-MB levels. Serum and plasma samples were prepared within 30 minutes of blood sampling in a precooled centrifuge and were immediately frozen and stored at 70°C until used for analysis. The assessment of cTnI blood levels was performed with Chiron Bayer ACS 180 chemiluminescent diagnostic test. This test is characterized by a high sensitivity, with a lower limit of detectability of 0.03 ng/mL. The upper limit of the normal cTnI value is 0.1 ng/mL.

Serum creatine phosphokinase isoenzyme MB (CPK-MB) activity level was measured by immunochemiluminometric assay (Chemilumi ACS, Centaur; Bayer Medical Co. Ltd., Tokyo, Japan), which have an upper reference limit of 5 ng/mL [14]. All parents signed a written informed consent before enrolment into the study. The local Institutional Research Ethics Committee approved the study protocol.

The power level of the number of cases in the study was more than 90%. Statistical analysis was performed with Statistical Package for Social Science (SPSS version 17). Data are presented as mean (±SD) values. Comparison between the studied groups was performed with Student's *t*-test, with *P* < 0.05 considered statistically significant. Wilcoxon's signed rank test was used to assess the normality of distributions of the data. The Bonferroni correction/adjustment procedure was done to avoid “significance” due to chance only, in multiple comparison with echocardiographic parameters. Correlation between variables was evaluated using Pearson's correlation coefficient [15].

## 3. Results

The demographics and clinical characteristics of children with ALL and the control group were shown in Table 2. There were no significant differences between children with ALL and the controls as regard to age and sex. The heart rate (HR) and respiratory rate (RR) were significantly higher in children with ALL than those in the controls (*P* < 0.001) while hemoglobin% (HB%), systolic blood pressure (SBP), diastolic blood pressure (DBP), and body mass index (BMI) were significantly lower in children with ALL than those in controls (*P* < 0.001).

Echocardiographic data of children with ALL and the controls before starting the doxorubicin treatment were shown in Table 3. The FS (which represents the LV systolic function) was significantly higher in leukemic children than that in the control group though still within the normal values (*P* < 0.05); however, the E/A (which represents the LV diastolic function) showed no significant differences between the 2 groups (*P* > 0.05). The table also showed that there were no significant differences in the tissue Doppler parameters: s, IVCT, *e*′, *a*′, *e*′/*a*′ ratio, and IVRT (*P* > 0.05). The global strain (*G*) of the LV was significantly lower in leukemic children than that in the control group (*P* < 0.05), and there was less significant decrease in the peak systolic strain in apical 2-chamber view (*P* < 0.05), while there was no significant change in the other views apical long axis and apical 4-chamber views (*P* > 0.05 for both views).

The echocardiographic examination data in the patient group before and after the doxorubicin treatment are shown in Table 4. It showed more significant reduction of FS after treatment with doxorubicin (*P* < 0.05). However the *s* wave measured by the tissue Doppler showed no significant difference before and after treatment. Meanwhile the IVCT showed significant prolongation after treatment. On the other hand, the diastolic function of the LV showed no significant differences both by conventional and tissue Doppler data except for IVRT which showed significant prolongation after the treatment with doxorubicin (*P* < 0.001). In 2D longitudinal strain echocardiogram, the peak systolic and global strains showed significant reduction in the apical long-axis view (*P* < 0.01 and <0.05, respectively, but showed no significant differences in both apical 4-chamber and apical 2-chamber views (*P* > 0.05).

**Figure 6:**
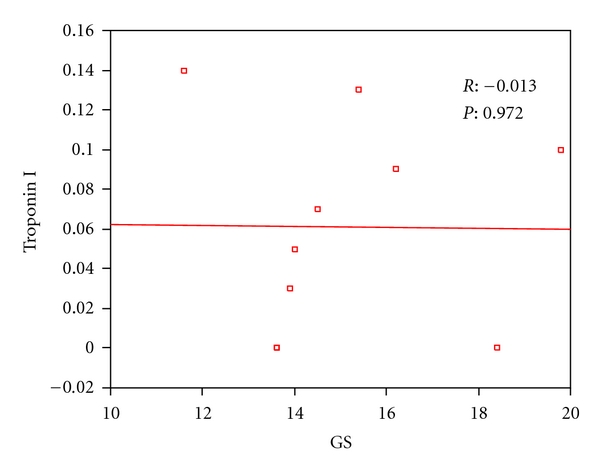
Correlation between troponin I and GS in ALL children after doxorubicin treatment.

**Figure 7:**
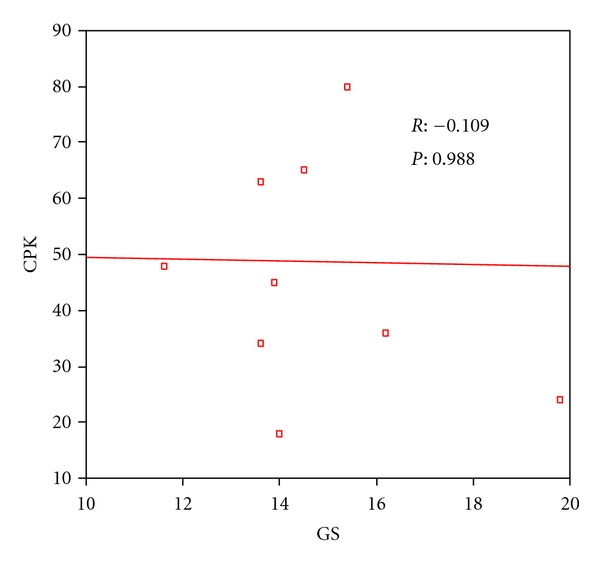
Correlation between CPK (MB) and GS in ALL children after doxorubicin treatment.

Table 5 showed significant increase in the serum cTnI level in the children with ALL after doxorubicin treatment than before starting the treatment. On the other hand, serum CPK (MB) level showed no significant change in those children after doxorubicin treatment. However, Figures 6 and 7 showed no correlation between the LV global strain (*G*) in the long axis view and both the cTn I and CPK (MB) level.

## 4. Discussion

Doxorubicin-induced cardiotoxicity is suggested to be through production of oxygen free radicals and highly reactive hydroxyl radicals and peroxynitrite which induce apoptosis and cardiac myocytes damage as the heart is particularly poorly protected against oxidative stress [16]. Other mechanisms implicated in the pathogenesis of doxorubicin-induced cardiotoxicity are lipid peroxidation, gene expression reduction, nucleic acid and protein synthesis inhibition, vasoactive amines release, adrenergic function alteration, calcium handling aberration, impairment of mitochondrial creatinine kinase, and induction of nitric oxide synthase [17]. Echocardiography is one of the most widely used noninvasive methods for early detection and monitoring of doxorubicin-induced cardiotoxicity. Left ventricular systolic function evaluation by measuring the ejection fraction or fractional shortening is the most commonly used method to early detect doxorubicin-induced cardiotoxicity either by nuclear methods or by echocardiography [17].

Early detection of doxorubicin cardiotoxicity is of paramount importance as it allows the use of cardioprotective agents dexrazoxane or carvedilol. In the current study, 3 echocardiographic modalities were used to early detect the doxorubicin-induced cardiotoxicity: the conventional 2D Doppler echocardiography by measuring the fraction shortening as well as the E/A ratio, the pulsed tissue Doppler by measuring the *s* wave, IVCT, *e*′ wave, *a*′ wave, *e*′/*a*′ wave, and IVRT, and lastly the 2D longitudinal strain echocardiography by assessment of LV longitudinal strain.

The fraction shortening showed a tendency towards becoming statistical significantly higher in the patients group when compared to the control. This difference can be explained by the presence of associated anemia and tachycardia secondary to leukemia (hyperdynamic heart). After doxorubicin treatment, the FS showed significant reduction despite still being within the normal limits. This reduction was either due to the correction of anemia prior to stating the cytotoxic therapy or as a side effect of therapy with doxorubicin. However, FS has many limitations as regard to the difficulty in accurate measurement and being affected by preload, after load, heart rate, desynchrony, as well as myocardial contractility [18]. The transmitral E/A ratio measured by conventional Doppler showed no significant difference between patients group and controls and also in the patients group before and after doxorubicin treatment. These findings disagreed with that of Iarussi et al. who found that E/A ratio was significantly reduced after doxorubicin treatment. This difference in the results could be because they studied the late cardiotoxicity after 1 year of doxorubicin treatment and after resolution of the acute stage of the disease [19]. However, presence of normal E/A ratio does not exclude presence of diastolic dysfunction as pseudonormal E/A ratio may be the case.

The pulsed tissue Doppler also showed no significant changes between the controls and the patients group and in the patient group before and after treatment except for the prolonged IVCT and IVRT after doxorubicin treatment which may indicate doxorubicin-induced impairment of LV relaxation and contraction. However, our findings disagreed with the work of Kapsuta et al. who found that the pulsed tissue Doppler was a useful sensitive method to detect subclinical myocardial damage in apparently healthy children who received moderate doses of anthracyclines for treatment of childhood malignancy [20]. This discrepancy could arise due to the long duration between starting the anthracyclines treatment and the timing of echocardiographic examination (within 5 years) as the severity of echocardiographic LV abnormalities increases with the duration of the followup [21]. However, the significant prolongation of IVCT and IVRT observed in our study may reflect beginning of impairment of the contractile and relaxation properties of myocardium and the inception of the cardiotoxicity.

On the other hand, the 2D longitudinal strain echocardiography was more sensitive to detect the early cardiac effects of leukemia and that of doxorubicin treatment, where there was significant reduction in the peak systolic stain in the apical long-axis view and in the global strain both between the patients group and the controls and in the patients group before and after doxorubicin treatment. Our findings agreed with a number of studies concerned with detection of cardiotoxic effects of doxorubicin and other anthracyclines. Migrino et al. studied doxorubicin-induced cardiotoxicity in 14 male Sprague-Dawley rats. They found that the global radial strain derived from 2-dimensional strain echocardiography was useful in the early detection of doxorubicin cardiac injury and that the reduction in radial strain was associated with the degree and the onset of histologic markers of doxorubicin-induced cardiomyopathy [17]. Tsai et al. investigated the long-term effect of anthracyclines on LV systolic function using two-D speckle tracking echocardiography. They found that the global longitudinal strain was reduced in patients receiving anthracyclines (doxorubicin 309 mg ±92) with mediastinal radiotherapy compared to the other group receiving mediastinal radiotherapy alone or combined radiotherapy and regimens without anthracyclines [22]. The same findings were observed by Piegari et al., who showed in a doxorubicin-induced cardiomyopathy model that strain and strain rate imaging were more sensitive indices in identifying early myocardial systolic changes induced by doxorubicin treatment than standard echocardiographic parameters and myocardial velocities [23]. They did the echocardiography at 2 and 4 weeks of treatment. However, the limits of studies of Migrino et al. and Piegari et al. were that these studies were performed on animal models and we cannot extend their data to the human being but they can be used as a guide.

What makes 2D strain echocardiography more sensitive than pulsed tissue Doppler is because of lack of the tethering effects from other myocardial segments which could limit the ability of tissue Doppler imaging to quantify regional function. The pulsed tissue Doppler directly measures the regional function rather than tissue velocities, which are also influenced by contractile function of other myocardial regions due to tethering. This could limit the ability of tissue Doppler velocities to provide quantitative data on regional function. However, combining both techniques can give complementary results (in our study, the prolonged IVCT and IVRT by pulse tissue Doppler and the reduction in the peak systolic and global strain by 2D strain echocardiography). However, marked angle dependency is a significant limitation of strain rate imaging, so that correct echo beam orientation is critical [24, 25].

## 5. Limitation of the Study

The study was performed before the induction phase and after the end of doxorubicin treatment. So the study did not check the late and chronic effects of doxorubicin treatment on the cardiac functions. The study also concentrated on the left side of the heart and did not evaluate the right side of the heart as well as for the pulmonary pressure. The study compared the 2D longitudinal strain echocardiogram with relatively older modalities that can assess the cardiac function. It did not compare the 2D longitudinal strain echocardiogram with cardiac magnetic resonance which is considered the gold standard for assessment of LV deformation. Also, there were many chemotherapeutic drugs used during the study. So, the cardiotoxic effects may be caused by doxorubicin or other cytotoxic drugs. However, due to ethical issues, we could not deprive a group of the patients from doxorubicin treatment to be included as a control group. The study also did not revise the effect of the cumulative doses of doxorubicin on 2D longitudinal strain echocardiogram. Another limitation of the study is that echocardiograms were not blindly read, so this could have induced some bias.

## 6. Conclusion

The 2D longitudinal strain echocardiography was more sensitive than conventional 2D and pulsed tissue Doppler echocardiography in detecting the early LV doxorubicin-induced cardiotoxicity in children with ALL. This is especially important to select the patients who need prophylactic therapy against doxorubicin-induced cardiotoxicity.

## Figures and Tables

**Table 1:** The protocol of induction of remission in children with newly diagnosed acute lymphoblastic leukemia in the Hematology and Oncology Unit, the Pediatric Department, Faculty of Medicine, Tanta University.

Drug	Intake route	Dose	Instructions
Vincristine	IV	1.5 mg/m^2^	Days 8, 15, 22, and 29
L-Asparaginase	IM	10000 IU/m^2^	3 times a week for 9 doses
Prednisone	PO	60 mg/m^2^/day	In TID: days 29–32
Prednisone	PO	30 mg/m^2^/day	In TID: days 29–32
Prednisone	PO	15 mg/m^2^/day	In TID: days 33–35, then discontinued on day 36
Triple therapy: Ara-C, methotrexate, and hydrocortisone	Intrathecal	Doses adjusted to the age	On days 1, 15, and 29
Conventional doxorubicin	IV	30 mg/m^2^	Days 8, 15, 22, 29

IV: intravenously, IM: intramuscularly, PO: orally, TID: on three divided doses daily.

**Table 2:** Comparison of demographic data in controls and ALL children group.

	Control (*n*-30)	Patient Group (*n*-25)	*t*-test	*P* value
Age ± SD (yr)	9.2 ± 2.9	9 ± 2.6	0.47	0.64
Sex M : F ratio	7 : 8	13 : 12	0.6	0.53
Hb% ± SD	12.8 ± 1.1	9.4 ± 1.05	10.2	<0.001*
HR ± SD (beat/min)	83.3 ± 8.0	87.0 ± 8.7	10.3	<0.001*
RR ± SD (cycle/min)	21.1 ± 2.5	22.4 ± 2.8	6.6	<0.001*
SBP ± SD (mmHg)	100.0 ± 6.2	90.8 ± 5.2	7.3	<0.001*
DBP ± SD (mmHg)	56.7 ± 6.3	49.2 ± 5.9	15.6	<0.001*
BMI ± SD (Kg/m^2^)	18.4 ± 1.7	22.0 ± 2.5	6.1	<0.001*

M : F (male-to-female ratio); Hb% (hemoglobin percent); HR (heart rate), RR (respiratory rate); SBP (systolic blood pressure) DBP (diastolic blood pressure); BMI (body mass index).

**Table 3:** Comparison between conventional echo, tissue Doppler parameters, and peak systolic strain in the main three longitudinal views of LV in controls and patients group before starting doxorubicin treatment.

	Control (*n*-30)	Patient group (*n*-25)	*t*-test	*P* value
FS %	35.78 ± 5.16	40 ± 4.87	2	0.05*
E (m/sec)	0.87 ± 0.11	0.77 ± 0.24	2.1	0.04*
A (m/sec)	0.52 ± 0.13	0.73 ± 0.13	3.3	0.005*
E/A	1.51 ± 0.4	1.60 ± 0.42	1.1	0.09
s (m/sec)	0.07 ± 0.02	0.06 ± 0.014	0.9	0.15
IVCT (ms)	83.1 ± 4.9	83.6 ± 4.2	1.8	0.08
*e*′ (m/sec)	0.12 ± 0.03	0.127 ± 0.011	0.5	0.52
*a*′ (m/sec)	0.07 ± 0.02	0.072 ± 0.020	0.8	0.25
*e*′/*a*′	1.88 ± 0.49	1.9 ± 0.4	0.75	0.53
IVRT (ms)	66.2 ± 3.6	67.1 ± 3.3	1.99	0.057
ALX	−22.2 ± 5.8%	−21.1 ± 5.3%	0.53	0.59
A4C	−21±2.4%	−18.9 ± 4.5%	1.86	0.07
A2C	−21±3.4%	−16.9 ± 7.3%	2.3	<0.03*
G	−21.5 ± 2.2%	−18.7 ± 4.5%	2.7	<0.01*

FS: fractional shortening, *E*: peak early filling velocity, *A*: Peak atrial phase filling velocity, *s*′: tissue Doppler peak mitral annulus systolic velocity, *e*′: tissue Doppler mitral flow early-phase filling velocity, *a*′: tissue Doppler peak atrial phase filling velocity, IVCT: isometric contraction time, IVRTL: Isometric relaxation time, ALX: apical long axis, A4C: apical 4-chamber, A2C: apical 2-chamber, views, and *G*: global peak systolic strain.

**Table 4:** Comparison between effects on patient group before and after doxorubicin on conventional echo and tissue Doppler parameters.

	Patient group before (*n*-25)	Patient group after (*n*-25)	*t*-test	*P* value
FS %	40 ± 4.87	33.5 ± 6.58	2.508	0.02*
E (m/sec)	0.77 ± 0.24	0.78 ± 0.24	0.214	0.83
A (m/sec)	0.73 ± 0.13	0.63 ± 0.13	1.244	0.32
E/A	1.60 ± 0.42	1.5 ± 0.37	4.4	1.06
s (m/sec)	0.063 ± 0.014	0.062 ± 0.01	0.18	0.56
IVCT (ms)	86.5 ± 4.2	85.9 ± 0.8	2.4	0.02*
*e*′ (m/sec)	0.127 ± 0.011	0.132 ± 0.009	1.099	0.26
*a*′ (m/sec)	0.072 ± 0.020	0.061 ± 0.011	1.468	0.52
*e*′/*a*′	1.852 ± 0.396	2.146 ± 0.373	1.708	0.105
IVRT (ms)	67.1 ± 3.28	7.1.8 ± 3.28	5.8	<0.001*
ALX	−21.13 ± 5.26%	−13.28 ± 3.69%	3.859	0.001*
A4C	−18.91 ± 4.51%	−17.27 ± 4.19%	0.841	0.41
A2C	−16.87 ± 7.25%	−14.75 ± 3.56%	0.829	0.42
G	−18.65 ± 4.52%	−15.10 ± 2.45%	2.182	0.04*

FS: fractional shortening, *E*: peak early filling velocity, *A*: Peak atrial phase filling velocity, *s*′: tissue Doppler peak mitral annulus systolic velocity, *e*′: tissue Doppler mitral flow early-phase filling velocity, *a*′: tissue Doppler peak atrial phase filling velocity, IVCT: isometric contraction time, IVRTL: Isometric relaxation time, ALX: apical long axis, A4C: apical 4-chamber, A2C: apical 2-chamber, views, and *G*: global peak systolic strain.

**Table 5:** Comparison between troponin I and CPK (MB) in patient group before and after doxorubicin.

	Patient group before (*n*-25)	Patient group after (*n*-25)	*t*-test	*P* value
Troponin I (ng/mL)	0.055 ± 0.003	0.061 ± 0.005	7.8	0.002*
CPK (MB) (U/L)	50.60 ± 8.55	48.61 ± 6.56	0.185	0.62

CPK MB: creatinine phosphokinase cardiac.
